# Development of a Generic Workshop Appraisal Scale (WASC) for Organizational Health Interventions and Evaluation

**DOI:** 10.3389/fpsyg.2020.02115

**Published:** 2020-08-18

**Authors:** Annemarie Fridrich, Georg F. Bauer, Gregor J. Jenny

**Affiliations:** ^1^Stiftung Patientensicherheit Schweiz, Zurich, Switzerland; ^2^Center of Salutogenesis, Division of Public and Organizational Health, Epidemiology, Biostatistics and Prevention Institute, University of Zurich, Zurich, Switzerland

**Keywords:** organizational health intervention, intervention research, evaluation research, process evaluation, process appraisal, outcome expectancy, scale development

## Abstract

This study presents the development of a generic workshop appraisal scale (WASC) for the evaluation of organizational health interventions. Based on the session evaluation questionnaire (SEQ) by Stiles (1980), we developed a short, generic 10-item scale with pairs of adjectives, covering five facets: comprehensibility, relevance, novelty, activation, and valence. Our study is based on *N* = 499 employees from four organizations who participated in 41 workshops and filled out an evaluation questionnaire on-site. The questionnaire contained the newly developed WASC, as well as items capturing satisfaction with the developed output and outcome expectancies. Results from confirmative factor analysis confirmed the hypothesized five-factor structure of the WASC. The factor structure was found to be nearly invariant across the four organizations, a result that needs to be replicated in larger samples. Analysis of intra-class correlations indicated that 25% of the variance in workshop appraisal can be explained at workshop level. Hereby, perceived relevance and novelty exhibited lower amounts of shared variance, indicating that corresponding workshop appraisals are influenced more by individual factors and less by group dynamics. Furthermore, results from mediation analysis revealed that participants’ workshop appraisals were significantly related to their outcome expectancies, and that this relationship was mediated by output satisfaction. Again, the facets showed differential effects: Relevance and comprehensibility seem to contribute most to the total effect on outcome expectancy, followed by activation, whereas valence and especially novelty play a minor role. Taken together, participants’ workshop appraisals – together with output satisfaction and outcome expectancy – may be helpful for monitoring the implementation process and allow for corrective action if necessary.

## Introduction

Organizational health interventions most commonly implement a series of participatory workshops to improve working conditions and evaluate the effects by means of pre-post surveys (see e.g., Biron et al., 2012; Nielsen and Noblet, 2018). As effect evaluations often revealed inconsistent results (Nielsen et al., 2010b), process and context evaluations have gained increasing attention to better understand the process of change (Biron and Karanika-Murray, 2014; Nielsen and Miraglia, 2017). One important focus of process evaluation is the implementation process, which can be defined as a “time-limited, actual enactment of all steps and elements of the original intervention plan” (Fridrich et al., 2015, p. 7). Hereby, process evaluation focusses on both the implementation of particular intervention elements (e.g., workshops, surveys, etc.) and of the overarching intervention architecture, that is, the realization of the complete project cycle. With regard to evaluating the implementation of particular intervention elements, researchers often capture factors such as “reach” and “dose,” for example, participant numbers, duration and frequency of elements or (self-reported) exposure to the intervention, often combined with perception of impact (Hasson et al., 2014; e.g., Biron et al., 2016). The overall implementation is often evaluated in terms of “fidelity,” answering the question if the implementation of the project cycle could be realized as intended (Augustsson et al., 2014). This has been combined with measures assessing the healthiness of the change process (Tvedt et al., 2009) or with general success factors of change derived from both occupational health intervention and management literature (Ipsen et al., 2015b; Jenny et al., 2015), interwoven with contextual factors that support or hinder change (Nielsen and Miraglia, 2017).

Meanwhile, all evaluation models in the field of occupational health psychology contain the assessment of process and context factors (Nielsen and Abildgaard, 2013; Nielsen and Randall, 2013; Fridrich et al., 2015; von Thiele Schwarz et al., 2016), whereby there is still much heterogeneity in regard to specific indicators (Havermans et al., 2016). It has also been stated that employees do not react passively to interventions, but rather actively craft and shape them (Nielsen, 2013), which also applies to the entire organization as dynamic social system (Jenny and Bauer, 2013; von Thiele Schwarz et al., 2016). In their systematic review, Murta et al. (2007) identified participants’ attitudes as one of the four most frequently used process evaluation factors in occupational stress-management programs, apart from recruitment, dose received and reach. Including participants’ attitudes toward intervention elements has shown that the more favorable the appraisal, the greater is the likelihood of positive work-stress related outcomes (Murta et al., 2007). This has been affirmed by other intervention researchers too (e.g., Flay et al., 2005; Nielsen et al., 2007; Randall, 2013) and process appraisals – with respect to the entire intervention and single intervention elements – have soon been captured in organizational health intervention research (e.g., Nielsen et al., 2007; Aust et al., 2010). Appraisal scales are commonly rated by the participants of the intervention, and they are labeled as ‘appraisals of interventions’ (Randall et al., 2009), ‘satisfaction with treatment’ (e.g., Brouwer et al., 2011; Joosen et al., 2011), ‘session evaluation’ (Busch et al., 2010), or ‘participants’ attitudes toward interventions’ (Murta et al., 2007). Taking a look at the concrete implementation process measures concerning particular intervention elements, one is confronted with a variety of mostly project-specific approaches. While some studies used quantitative instruments (e.g., Nielsen et al., 2007; Strijk et al., 2011; Lien and Saksvik, 2016; Dollard and Zadow, 2018), others used qualitative instruments (e.g., Konradt, 2000; Busch et al., 2010), and still others combined quantitative and qualitative evaluations (e.g., Veach et al., 2003; Augustsson et al., 2014; Abildgaard et al., 2016). A multi-method approach to process evaluation is generally recommended to capture the implementation of both single elements and the overall architecture. Thus, to facilitate quantitative process evaluation of contextualized projects in heterogenous companies, we aimed to develop and test a short, generic scale for workshop evaluations.

Some researchers suggested that it might be valuable to use and adapt concepts and ideas from other disciplines, such as psychotherapy, as this discipline in particular “is somewhat ahead of SMI [stress-management interventions] work in its development” (Bunce, 1997, p. 2). For instance, Busch (2004) successfully used elements of the session evaluation questionnaire (SEQ) by Stiles (1980), which was originally developed for measuring the impact of psychotherapy sessions, to build a scale measuring the mood of participants after engaging in stress-management training for teams. The SEQ has the advantage of being unspecific in regard to content, capturing session appraisals with pairs of adjectives. Yet as the SEQ was designed to capture the interaction between a client and a therapist, the scale couldn’t be applied one to one to the organizational setting. For example, some adjectives capture characteristics of the therapeutical setting (such as “safe vs. dangerous”), which don’t fit well to the kind of interventions conducted in organizational health interventions. Thus, based on the approach underlying the SEQ and the range of its adjectives, the present paper develops a short, generic scale to capture process appraisals of intervention elements. As these predominantly have workshop character in organizational health interventions, we label the scale ‘workshop appraisal scale (WASC).’ A preliminary version of the WASC was applied in the evaluation of a participatory intervention in a large hospital (see sections “Materials and Methods” and “Discussion”; Füllemann et al., 2016).

We postulate five facets being worth captured during the implementation process: (1) comprehensibility, (2) relevance, (3) novelty, (4) activation, and (5) valence. We elaborate the selection of these facets as follows:

(1) *Comprehensibility.* Findings from health education research have demonstrated that the comprehensibility of a program or educational material is an essential precondition for the success of health promoting activities (Bauman, 1997; Farin et al., 2013). The SEQ too includes an item for measuring the difficulty of the session (difficult vs. easy session). Applied to organizational health interventions, the implemented workshops should be perceived as comprehensible and clear.
(2) *Relevance.* An intervention element can only be of benefit when the topics covered are relevant to the participants. Researchers have emphasized the importance of perceived relevance or person-intervention fit (e.g., Nielsen et al., 2007; Randall and Nielsen, 2012). Some researchers have included measures that capture the extent to which an intervention fits the participants’ needs. For example, Linna et al. (2011) investigated the connectedness of intervention activities to everyday work. Individual effectiveness varies between different participants, depending on the fit between the delivered intervention and an individual’s needs (Kompier and Kristensen, 2000). The SEQ includes an item that measures how valuable a psychotherapy session was perceived to be by the patient (valuable vs. worthless session). Thus, despite the fact that a workshop may be perceived as comprehensible, it may not produce the desired effect if it lacks importance and relevance for the participating individuals.
(3) *Novelty.* This facet suggests that an intervention element must convey some innovative content to be effective. The terms innovativeness and degree of novelty are mostly discussed in the context of product development and marketing. A few authors address them within other contexts. For instance, Isaksen and Tidd (2006) described the degree of novelty as an important success factor that is relevant to the process of change in organizations. In the context of adult education, Wlodkowski and Ginsberg (2017) argued that novelty facilitates the attraction of students’ attention. This is in line with research on motivational behavior, which demonstrates that individuals strive for situations with an adequate level of novelty, that is, representing a learning challenge while still being manageable, resulting in a high degree of self-engagement (Csikszentmihalyi, 1990). These findings illustrate that the perceived degree of novelty can act as an activating and motivating factor. The SEQ includes an item that measures the peculiarity of a session (ordinary vs. special session). Based on this research, we hypothesize that workshops in occupational health interventions should also be special and new to the participants.
(4) *Activation.* This facet captures the extent to which an individual is activated in a workshop and can put forward her/his own experiences, opinions, and needs. The concept of participation is one of the most recognized and well-researched success factors in organizational health interventions and evaluations (von Thiele Schwarz et al., 2016). Approaches that are perceived as participative by the target group have advantages over non-participatory interventions (Nytrø et al., 2000; e.g., Linna et al., 2011): For instance, Nielsen et al. (2007) found that the perceived opportunity to influence an intervention project correlates with the overall process appraisal of an intervention. According to Nielsen et al. (2010a), employee participation has three essential advantages: First, it facilitates access to employees’ specific job expertise and knowledge. Second, it works as an intervention itself, because the mere involvement of participants activates various resources, such as increased control, respect, and justice perception. And third, it has a positive influence on the change process, particularly on resistance to change. Such participation and involvement in workshops should trigger a sense of activeness and vividness versus feeling passive and experience monotony. As the SEQ was developed for small-group therapeutical sessions, there are no template items.
(5) *Valence.* The final facet refers to the affective appraisal of an intervention element. This overall impression is based on the participant’s current mood and previous experience with interventions. Furthermore, the general attitude toward the intervention project and the emotional appraisal of the group’s atmosphere might shape this impression. The overall affective impression can be described as a multifaceted and uncontrollable emotion that influences the effectiveness of an intervention element. According to Castelfranchi (2000), this emotion can stimulate individuals to identify goals – for example, if a workshop triggers pleasurable feelings in a person, such as joy and confidence, it is likely that this person will set individual goals on the basis of the workshop and engage in the workshop activities as well as subsequent actions of change. The SEQ includes a valence item (bad vs. good session), which might be applied to organizational health interventions too.

Besides the appraisal of intervention elements such as workshops, we propose to capture two further factors during implementation: *outcome expectancies* and *output satisfaction*. Outcome expectancies can be “defined in terms of participants’ assumptions about the consequences of an intervention element” (Fridrich et al., 2016, p. 6). Several researchers have studied outcome expectancy in the context of individuals’ behavioral changes and found significant relationships between the outcome expectancy of a project, activity, or behavior and the following: responsibility for a project (Feather et al., 2012), willingness to support an activity (Feather and Newton, 1982), behavioral intentions (Maddux et al., 1982), and outcome behaviors (Resnick et al., 2000). Bunce (1997) argued that it is likely that such expectations moderate the effectiveness of an intervention. Fridrich et al. (2016) found that the outcome expectancy of a stress-management workshop is able to partly predict the perceived individual and organizational impact of the entire stress-management intervention in a 2-year follow-up. Similarly, both Füllemann et al. (2016) and Lehmann et al. (2019) showed that outcome expectancy – measured during workshops of an organizational health intervention – was related to follow-up perceptions of impact. Assuming that an individual’s outcome expectancy reflects their assessment of whether changes triggered by an intervention element will influence their well-being, the outcome expectancy will influence the individual’s decision to participate (or not to participate) in activities developed during the intervention element and thus, influence the intervention’s impact.

Organizational health interventions aim to produce concrete output, such as survey results, a list of activities, task allocations or regulations in written form, or even drawn images developed by participants during a workshop (Bauer and Jenny, 2018). Examples are Kaizen workshops (Augustsson et al., 2014; von Thiele Schwarz et al., 2017), Fishbone workshops (Ipsen and Andersen, 2013; Ipsen et al., 2015a), Future workshops (Bauer et al., 2014) or Health circles, all of which enable participants to identify job demands and resources and develop concrete measures to improve them (Aust and Ducki, 2004). This output plays an important role for the effectiveness and success of the intervention. A recent study showed that workshop output in form of if-then plans – together with outcome expectancies – predicted the perceived impact of an intervention (Lehmann et al., 2019). This applies not only to the output’s form, but also its appraisal: Linna et al. (2011) implemented a participatory intervention on organizational justice perceptions where various work groups developed and implemented tailored action plans according to their specific groups’ needs. They used five items to evaluate the implementation of the intervention from the participants’ views, with one item explicitly referring to the appraisal of the output. They found significant associations between the appraisal and various outcome measures, such as improvements in justice perception.

In the present study, we developed the WASC with 10 adjective pairs (see measures) and combined it together with measures of outcome expectancy and output satisfaction in an evaluation questionnaire. Latter was applied in a range of workshops implemented as part of several occupational health interventions. We formulated five hypotheses as part of the WASC development.

(H1) We presume that the WASC facets comprehensibility, relevance, novelty, activation, and valence are correlated, but that they are distinguishable. We hypothesize: *The facets of comprehensibility, relevance, novelty, activation, and valence can be distinguished in confirmatory factor analysis (Hypothesis 1).*
(H2) The WASC was developed to be non-content specific and thus applicable to different interventions in diverse organizations. Therefore, we hypothesize the following: *The factor structure of the WASC is invariant across organizations (Hypothesis 2).*
(H3) When workshop participants work together on individual and common themes, planned and unplanned group processes and social dynamics often emerge to different degrees (Karanika-Murray and Biron, 2013). These processes and dynamics influence individuals’ appraisals of the respective workshops. Given that the workshop appraisals incorporate portions from individuals’ perceptions of the workshops and portions from shared appraisals, we suppose the following: *The participants’ workshop appraisals are more similar within workshops than between workshops (Hypothesis 3).*
(H4) Although outcome expectancy might be influenced by a number of individual and organizational factors, such as individual commitment or manager support, it seems reasonable that participants who rate a workshop as favorable are also more confident that the workshop will have positive outcomes. Thus, we assume the following: *Participants’ workshop appraisals are positively related to their outcome expectancies (Hypothesis 4).*
(H5) If participants rate the workshop as favorable, it is likely that they had the opportunity to contribute to the workshop – and in particular – to the development of output in form of action plans, or similar. As outlined above, studies showed that output appraisal as well as outcome expectancies are related to perceived intervention impact (Linna et al., 2011; Fridrich et al., 2016; Füllemann et al., 2016). From this we suppose that output satisfaction strengthens trust in the efficacy of the workshop and the action plans, which will impact outcome expectancies as potential success factor of interventions. Thus, we assume the following: *Participants’ output satisfaction partially mediates the relationship between participants’ workshop appraisals and participants’ outcome expectancies* (*Hypothesis 5).*

## Materials and Methods

### Intervention

The WASC was developed to evaluate organizational health interventions in four different companies in the German-speaking part of Switzerland. The projects were initiated in 2013 and lasted until 2016.

*Organization one* is a large hospital that implemented a project focusing on the introduction of lean-principles in all nursing wards in the hospital. For each ward, a 4-day lean-workshop with representatives from each nursing level took place. During the lean-workshop, the participants discussed the results of an employee survey and developed action plans to improve their work situation. Apart from lean-management, the workshop covered psychosocial working conditions, such as work-life balance, team climate, job demands and resources, and interprofessional collaboration between nursing staff and physicians. The workshop was evaluated at the end of the second and fourth workshop days. For this study, only evaluation data from the second day were included, when action plans were developed. The fourth workshop day served as a refresher workshop (see below too).

*Organization two* operates in the field of constructing, servicing and maintaining energy and telecommunication networks. A work-life balance intervention was implemented separately at each of the two branches: It comprised an introductory workshop in which the results of an employee survey were presented to the leaders, a skill-development workshop for leaders, and a team workshop. The skill-development workshop for leaders aimed to enhance health-related knowledge and leaders’ skills, while also empowering them to implement workshops with their own teams. The team workshops covered an analysis of the work and health situation of the respective teams, as well as the development of action plans for strengthening job resources and reducing job demands. Refresher workshops for leaders and teams took place 6 months after the primary workshops. For this study, only evaluation data of the primary workshops were included, as various measures were implemented subsequently, influencing appraisals in the refresher workshops.

The same intervention architecture was applied to *organization three*, a mail-order pharmacy, and to organization *four*, a public administration, whereby the survey results-workshop was integrated into the skill-development workshop for leaders. Due to company size, the intervention was implemented separately in two functionally similar areas of organization *four*, as it was done in organization two. Taken together, the WASC was applied to four types and a total of 41 workshops (see Table 1).

**TABLE 1:** Composition of the study sample concerning workshoptype and organizational affiliation.

Workshop type	Organization 1	Organization 2	Organization 3	Organization 4	Total
Survey results-workshop		33 (2)			33 (2)
Leaders workshop		30 (2)	17 (1)	23 (2)	70 (5)
Team workshop		109 (2)	49 (1)	81 (2)	239 (5)
Lean workshop	157 (29)				157 (29)
Total	157 (29)	172 (6)	66 (2)	104 (4)	499 (41)

*Values in brackets display the number of workshops.*

### Sample

Nine hundred and thirty-three evaluation questionnaires from 74 workshops were returned to our research group, whereof 335 questionnaires covered the refresher workshops and were thus excluded for this study, resulting in a total of 598 questionnaires eligible for this study. Another 66 questionnaires were excluded due to missing data in the WASC. We checked the WASC data for skewness (largest value: 1.506), kurtosis (largest value: 2.529) and collinearity (no correlation above 0.80). Further we calculated Mahalanobis values to check the WASC data for multivariate outliers; based on the cut-of criteria for df = 10 and *p* < 0.001 (29.588), we excluded 33 cases. Our final study sample is *N* = 499 from 41 workshops. The average number of workshop participants was 13 (Median = 6, Min = 2, Max = 58). Table 1 provides detailed information concerning the composition of the study sample. To test hypotheses on the relationship between WASC, output satisfaction and outcome expectancy, a reduced sample of *n* = 394 participants was used due to missing values of latter variables and due to the fact that output satisfaction was not captured in some of the workshops, as no action plans were developed. Demographic information about the participants was not captured in the questionnaire to ensure complete anonymity (see section “Procedure” below).

### Procedure

All workshops were evaluated with the same evaluation questionnaire. Participants voluntarily completed the two-page workshop evaluation questionnaire in the presence of the respective workshop instructor(s) at the end of the workshop. Completed questionnaires were directly returned to the research group using pre-stamped reply envelopes. The overall response rate for the workshop evaluation questionnaire was 91% (ranging from 86% to 100% across intervention projects). No ethical review was necessary under national, university or departmental rules. The study was conducted under strict observation of ethical and professional guidelines.

### Measures

#### Workshop Appraisal Scale (WASC)

The WASC was developed as a generic, short 10-item scale (see Table 2) that can be applied with little effort in intervention studies facing limitations to questionnaire length. Each of the five facets was represented by two items. Items of the SEQ (Stiles, 1980) served as template (see section “Introduction”) and were complemented with further pairs of adjectives. Initially, for each facet a set of five pairs was developed. These were reduced to two pairs per facet based on pre-tests within the research group (as pre-tests within the companies were not feasible), ranking what items captured the corresponding facets best. The items were then shuffled and recoded: Comprehensibility (Items 1r + 6r), relevance (Items 2 + 7), novelty (Items 3 + 8), activation (Items 4 + 9), and valence (Items 5r + 10r). Referring again to the SEQ, we used a 7-point semantic differential format. Participants were asked to state how they personally perceived the workshop and to mark a cross in each row at the point that best corresponded with their feelings (see exact wording in Table 2). The WASC was applied in German and revealed a Cronbach’s alpha of 0.889 (0.857/0.899/0.839/0.870 for the respective organizations 1 to 4; for the inter-item correlations of the facets see Table 3).

**TABLE 2:** The workshop appraisal scale (WASC).

*How did you personally perceive the workshop?* *(Put a cross in each row at the point which best corresponds* *with your feelings.) I perceived the workshop as*…									

Item No.		Very		Neither			Very		
1	Comprehensible	○_1_	○_2_	○_3_	○_4_	○_5_	○_6_	○_7_	Incomprehensible
2	Unimportant	○_1_	○_2_	○_3_	○_4_	○_5_	○_6_	○_7_	Important
3	Ordinary	○_1_	○_2_	○_3_	○_4_	○_5_	○_6_	○_7_	Special
4	Monotonous	○_1_	○_2_	○_3_	○_4_	○_5_	○_6_	○_7_	Vivid
5	Positive	○_1_	○_2_	○_3_	○_4_	○_5_	○_6_	○_7_	Negative
6	Clear	○_1_	○_2_	○_3_	○_4_	○_5_	○_6_	○_7_	Unclear
7	Irrelevant	○_1_	○_2_	○_3_	○_4_	○_5_	○_6_	○_7_	Relevant
8	Well-known	○_1_	○_2_	○_3_	○_4_	○_5_	○_6_	○_7_	New
9	Passive	○_1_	○_2_	○_3_	○_4_	○_5_	○_6_	○_7_	Active
10	Good	○_1_	○_2_	○_3_	○_4_	○_5_	○_6_	○_7_	Bad

*Item 1, 5, 6, and 10 need to be recoded for the calculation of the scale value (see section “Limitations”).*

**TABLE 3:** Means, standard deviation, skewness, kurtosis, and correlations for the 10 WASC items.

Item no.	*M*	*SD*	Skew.	Kurt.	1	2	3	4	5	6	7	8	9	10
1. Comprehensible (C)	5.94	1.07	−1.28	−1.28	–									
2. Important (R)	5.76	1.34	−1.32	−1.32	0.39	–								
3. Special (N)	5.25	1.17	−0.59	−0.59	0.32	0.56	–							
4. Vivid (I)	5.61	1.21	−1.04	−1.04	0.46	0.54	0.63	–						
5. Positive (V)	5.75	1.29	−1.38	−1.38	0.54	0.36	0.39	0.41	–					
6. Clear (C)	5.65	1.21	−1.08	−1.08	0.61	0.32	0.31	0.38	0.72	–				
7. Relevant (R)	5.79	1.13	−1.16	−1.16	0.45	0.63	0.57	0.62	0.42	0.32	–			
8. New (N)	5.37	1.35	−0.72	−0.72	0.16	0.37	0.43	0.33	0.20	0.12	0.40	–		
9. Active (I)	5.78	1.13	−1.11	−1.11	0.45	0.54	0.57	0.68	0.40	0.39	0.62	0.34	–	
10. Good (V)	5.97	1.01	−1.16	−1.16	0.60	0.52	0.48	0.50	0.58	0.55	0.51	0.26	0.55	–

*N = 499. All correlations are significant at p < 0.05. Items 1, 5, 6, and 10 have been recoded. C, comprehensibility; R, relevance; N, novelty; I, activation; V, valence.*

#### Output Satisfaction

The workshop evaluation questionnaire included one item to capture satisfaction with action plans that were developed during the workshop (“Overall, how satisfied are you with the developed action plans?”) (Füllemann et al., 2016). The items were rated by the workshop participants on a 7-point Likert scale from 1 = no, not at all to 7 = yes, very much.

#### Outcome Expectancy

The workshop evaluation questionnaire included the following two items concerning the outcome expectancy of the participants (“Do you think the workshop will have a positive impact on your work?”; “Do you think the workshop will have a positive impact on your team?”) (Fridrich et al., 2016). The items were rated by the workshop participants on a 7-point Likert scale from 1 = no, not at all to 7 = yes, very much. The inter-item correlation was 0.69, *p* < 0.001 (0.71/0.68/0.67/0.69 for the respective organizations 1 to 4).

### Statistical Analyses

To test hypothesis 1, a confirmatory factor analysis (CFA) was conducted. Factorial invariance across the organizations (hypothesis 2) was tested using multiple group analysis. This technique is based on a comparison of the default model with a constrained model: In our study, we calculated a first model with equality constraints on all factor loadings and a second model adding equality constraints on factor variances and covariances. Differences in fit indices are used as invariance indicators. We used the difference in the root mean square error of approximation (RMSEA) and the difference in the comparative fit index (CFI) as decision criteria. According to Chen (2007), a ΔRMSEA ≥ 0.010 and ΔCFI ≥ 0.005 indicate non-invariance in smaller samples (total *N* < 300) and unequal sample sizes. Latter can be observed in our study (see Table 1), so we decided to apply these conservative cut-off criteria. Hypothesis 3 was tested by calculating the ratio of the between-group variance to the total variance [τ/(τ + σ2)], called intra-class correlation (ICC) (Woltman et al., 2012). In this study, the ICC represents the percent of shared variance within the workshops. A value of 1% shared variance is considered as small, 10% as medium and 25% as large (LeBreton and Senter, 2008). To investigate the relationship between the WASC, output satisfaction and outcome expectancies (Hypotheses 4 and 5), we calculated a path model with the observed variables and conducted mediation analysis with bootstrapping (5000 resamples). IBM SPSS Statistics 25 for Mac and the AMOS 23 software package were used for these analyses.

## Results

### Descriptive Results

Table 3 displays the correlations of the 10 WASC items of the appraisal scale. Correlations range between 0.12 and 0.68 and are all significant at *p* < 0.05. Furthermore, we found that output satisfaction (*M* = 5.63, *SD* = 1.03) and outcome expectancy (*M* = 5.86, *SD* = 1.07) are significantly intercorrelated (*r* = 0.64, *p* < 0.001) (see Table 6).

### Testing of Hypotheses

#### Factorial Structure of the WASC

Results of the CFA revealed that the five-factor solution (Model 2a) fits the data better than the one-factor (Model 1a). Table 4 shows the fit-indices. Studying the modification indices we found that the error terms of the four items that had to be recoded (Item 1, 5, 6, and 10) seemed to be highly correlated, especially those of items 5 and 6 that follow each other in the questionnaire. Thus, we tested two further models where the error terms of the items 5 and 6 were correlated (Model 1b + 2b), revealing a superior fit of the five-factor solution to the data. Finally, we also tested a one-factor solution where all errors of the recoded variables were allowed to correlate (Model 1c). This model had a poorer fit than Model 2b, which indicates support for hypothesis 1.

**TABLE 4:** Fit indices for the 1 and 5 factor models.

Model	χ^2^	*df*	CFI	RMSEA	LB 90	UB 90
Model 1a (1 factor)	534.464	35	0.805	0.169	0.157	0.182
Model 1b (1 factor + error corr. items 5/6)	321.496	34	0.888	0.130	0.118	0.144
Model 1c (1 factor + error corr. items 1/5/6/10)	97.256	29	0.973	0.069	0.054	0.084
Model 2a (5 factors)	133.667	25	0.958	0.093	0.078	0.109
Model 2b (5 factors + error corr. items 5/6)	49.607	24	0.990	0.046	0.028	0.065

*N = 499. χ^2^, chi-square statistic; df, degrees of freedom; CFI, comparative fit index; RMSEA, root-mean square error of approximation; LB, lower bound confidence interval of RMSEA; UB, upper bound confidence interval of RMSEA. All chi-square coefficients are significant at p < 0.01.*

The results of invariance testing (see Table 5) partially support hypothesis 2: Factor loadings are invariant over the four organizations, whereas equality constraints on the factor variances and covariances result in a ΔCFI of −0.030 which is above the cut-off value of 0.005; on the other hand, ΔRMSEA is 0.008 and thus below the cut-off value of 0.010.

**TABLE 5:** Invariance of the process appraisal scale across the four organizations (Model 2b).

Model	χ^2^	*df*	CFI	RMSEA	LB 90	UB 90
Unconst. model	219.316	137	0.965	0.035	0.026	0.043
Const. Model 1 (factor loadings)	232.469	147	0.964	0.034	0.026	0.042
Const. Model 2 (+factor variances and covariances)	333.715	177	0.934	0.042	0.041	0.054
Const. 1 vs. Unconst. (factor loadings)	Δχ^2^ = 13.153^*a*^	Δ*df* = 10	ΔCFI = −0.001	ΔRMSEA = −0.001	–	–
Const. 2 vs. Const. 1 (+factor variances and covariances)	Δχ^2^ = 101.246^*b*^	Δ*df* = 30	ΔCFI = −0.030	ΔRMSEA = 0.008	–	–

*N = 499. χ^2^, chi-square statistic; df, degrees of freedom; CFI, comparative fit index; RMSEA, root-mean square error of approximation. ^a^p = 0.215, ^b^p < 0.001.*

#### Shared Variance on Workshop Level

To test hypothesis 3, the WASC was computed as mean value from all 10 items. An analysis of variance for the overall scale showed that on individual level variance was σ2 = 0.555 and on workshop level variance was τ = 0.188, which results in an ICC of 0.253. This indicates that 25.3% of the workshop appraisal variance is at the workshop level, which can be considered as large and thus supports hypothesis 3. ICC was also computed for the WASC facets, revealing differences in shared variance: Comprehensibility has 22.3% shared variance, relevance 12.3%, novelty 9.2%, activation 25.8%, and valence 19.8%.

#### Relations With Output Satisfaction and Outcome Expectancies

Correlations showed that both the WASC overall scale as well as the WASC facets are significantly related to outcome expectancies, which supports hypothesis 4 (see Table 6).

**TABLE 6:** Means, standard deviation, skewness, kurtosis, and correlations for the five WASC subscales, the overall WASC scale, output satisfaction and outcome expectancy.

Scale no.	*M*	*SD*	Skew.	Kurt.	1	2	3	4	5	6	7	8
1. WASC Comprehensibility	5.90	0.98	−0.93	0.35	–							
2. WASC Relevance	5.82	1.10	−1.17	1.06	0.48	–						
3. WASC Novelty	5.35	1.08	−0.61	−0.02	0.30	0.62	–					
4. WASC Activation	5.80	1.05	−1.32	1.93	0.50	0.74	0.60	–				
5. WASC Valence	5.95	0.99	−1.09	0.81	0.77	0.54	0.39	0.54	–			
6. WASC Overall	5.76	0.83	−0.72	−0.08	0.75	0.85	0.74	0.85	0.80	–		
7. Output satisfaction	5.63	1.03	−0.79	0.52	0.39	0.44	0.32	0.41	0.40	0.49	–	
8. Outcome expectancy	5.86	1.07	−0.98	0.79	0.48	0.45	0.31	0.45	0.47	0.54	0.64	–

*N = 394. All correlations are significant at p < 0.001.*

**FIGURE 1:**
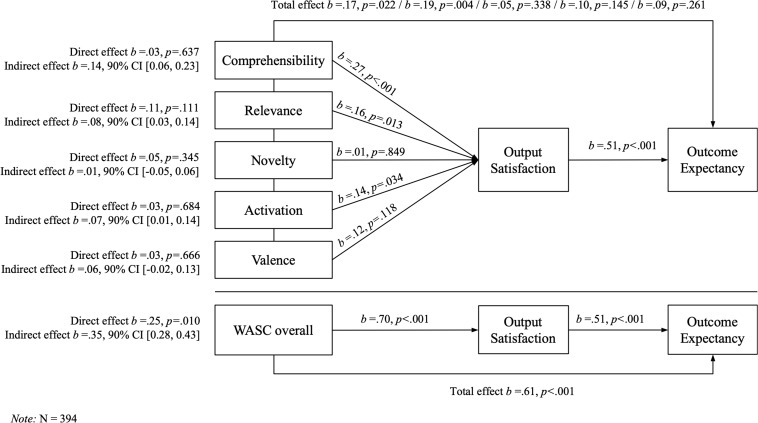
Mediation analyses with the WASC facets and the WASC overall value, respectively, as independent variables, with output satisfaction as mediator and outcome expectancy as dependent variable.

Results from mediation analysis revealed that workshop appraisal is indirectly related to outcome expectancies through its relationship with output satisfaction. As can be seen in Figure 1 (bottom, WASC overall), participants who positively assessed the workshop also reported more satisfaction with its output (*b* = 0.70, *p* < 0.001). More satisfaction with the workshop output was subsequently related to more positive outcome expectancies (*b* = 0.51, *p* < 0.001). Confidence intervals indicated an indirect effect of *b* = 0.35 (90% CI [0.28, 0.43]), supporting hypothesis 5 that workshop appraisal is related to outcome expectancy through output satisfaction. As hypothesized, this mediation is partial: Participants who positively assessed the workshop reported more positive outcome expectancies (*b* = 0.25, *p* = 0.010) even after taking into account the indirect effect via output satisfaction.

The same analysis was conducted with the WASC facets (see again Figure 1), revealing differential effects: The effect of comprehensibility on outcome expectancy was mainly indirect via output satisfaction (*b* = 0.14, 90% CI [0.06, 0.23]). The same applied to activation and to relevance, although the latter revealed a direct effect of *b* = 0.11 and was significant on *p* < 0.15. The indirect effect of valence was marginally non-significant, and novelty had neither a direct nor an indirect impact on outcome expectancy. We additionally estimated curvilinear relationships between the WASC facets and both output satisfaction and outcome expectancies, which didn’t prove to explain more variance than linear models.

## Discussion

This paper presented the development of a short, generic 10-item scale for measuring the appraisal of workshops (WASC) in the context of organizational health interventions. The WASC covers five facets: comprehensibility, relevance, novelty, activation, and valence. The single items were developed on the basis of an existing instrument from psychotherapy research, that is, the SEQ by Stiles (1980). Results confirmed that the hypothesized five-factor structure of the WASC could be distinguished. Further, the factor loadings seem to be invariant across the organizations, but not the factor variances and covariances. Differences in factor variance and covariance may be attributable to sample size, but also to the structure of the organizations and samples, respectively. For example, organization one consisted exclusively of nursing staff, whereas organization two consisted of both blue and white collar workers engaged in wider range of tasks. Additionally, some of the organizations were more experienced with conducting workshops and reflecting on their work by means of questionnaires, all of which may have an impact on the observed variance.

Further, we found that workshop appraisals were more similar between participants who attended the same workshop than between participants who attended different workshops. Hereby, perceived relevance and especially novelty exhibited lower amounts of shared variance, indicating that corresponding workshop appraisals are influenced more by individual factors and less by group dynamics. The group dynamics in such workshops have been little researched so far. As Karanika-Murray and Biron (2013) proposed, such dynamics may encompass mechanisms of social learning and comparison, identity and meaning building, as well as interpersonal influence, diffusion, contagion, and spillover effects. Such social mechanisms could explain larger proportions of shared variance in facets like comprehensibility (through social learning and comparison) or activation and valence (through contagion and spillover effects, for example) – which may also be amplified by an attentive workshop moderator. Facets like novelty on the other hand may be more dependent on the individual’s history of participation in such workshops and knowledge of occupational health. Yet a facet like relevance could be influenced through social identity and meaning building processes, where the workshop activities are collectively integrated into the group’s goals and values (see practical implications too). In the current study, with 12.3% shared variance, this seems to have been only partly the case (Inauen et al., 2017).

The results also support the assumption that participants who appraise a workshop as favorable also have high outcome expectancies. This relationship is partially mediated by satisfaction with the output developed during a workshop: Participants who rate a workshop as favorable are also more likely to be satisfied with the developed action plans, which in turn leads to high outcome expectancies: Outcome expectancies are known to be related to the perception of intervention success (Fridrich et al., 2016; Lehmann et al., 2019). A study utilizing a preliminary, not yet-validated version of the WASC aggregated on team level revealed that outcome expectancies – rather than workshop appraisals – were directly relevant for changes in working conditions, but also indicated that positive appraisal of the workshops was related to changes in affective activation at work (Füllemann et al., 2016). The mediating effect of output satisfaction between workshop appraisal and outcome expectancy might be caused by the fact that participants who are highly involved during the workshops also perceive the opportunity to contribute to the development of action plans and shape them according to their needs and context, which results in higher satisfaction concerning these plans. This high satisfaction is likely to strengthen their trust in the efficacy of the action plans, which becomes apparent in high outcome expectancies for the workshop. Lehmann et al. (2019) showed for example that if-then plans – i.e., the workshop output in form of concrete action plans – were related to the perception of intervention success.

Our mediation analysis also revealed differential effects of the WASC facets on outcome expectancy: Relevance and comprehensibility seem to contribute most to the total effect on outcome expectancy, followed by activation, whereas valence and especially novelty play a minor role. That novelty had little explanatory power in regard to output satisfaction or outcome expectancies was surprising, as its relevance for motivation (Csikszentmihalyi, 1990) or meaning (Heintzelman and King, 2014) has been discussed by various authors from different fields of research (see section “Introduction” too). From this we may hypothesize that novelty plays less a role for output, outcome or impact appraisals, but that it rather acts as a momentary factor interplaying with the experience of relevance and activation in a workshop (see correlations in Table 6). That is, during an active, participatory workshop new insights might emerge, leading to actions that are perceived as relevant. Such sparks of recognition could serve as drivers of the workshop and the development of output, but less of the subsequent change process. Last but not least it must also be recognized that organizational health interventions are seldom transformational or (radically) innovating processes, but rather incremental change within the routines and logics of the system (Jenny and Bauer, 2013).

### Limitations

Each facet of the process appraisal was represented by only two facets, as we were not able to test more items due to the organizations’ limits concerning the length of the workshop evaluation questionnaire. The superior fit of the five-factor solution was achieved by allowing correlation of two errors. The correlation of errors seems reasonable as only error terms of items that needed to be recoded and were next to each other in the questionnaire were allowed to correlate. According to Anderson and Gerbing (1988) “consideration of theory and content both […] reduces the possibility of taking advantage of sampling error to attain goodness of fit” (p. 416) and thus, we assess the procedure as legitimate. In future research, however, the direction of items could be either randomly varied or presented in one direction only. Similarly, the items could be presented in a random order. Further, the results of the invariance testing must be replicated with larger study samples in future research. According to Meade and Bauer (2007), it is difficult to detect invariance when the group size is small. Although the number of cases in three of four organizations was above 100, the statistical power of the invariance testing might be considered rather low (cf. Kline, 2016). Finally, the present study is based on cross-sectional data, as all scales captured momentary perceptions of the intervention process and workshop, respectively, which cannot be separated easily.

### Practical Implications and Future Research

For process evaluation of organizational health interventions, we recommend to report the WASC as overall value plus the values of the five facets. Given that high perceived quality of implementation and high outcome expectancies have been proven to be associated with positive intervention outcomes, process data on participants’ workshop appraisals together with their output satisfaction and outcome expectancies could be helpful for monitoring the implementation process and allows for corrective action in this process when necessary. Workshop moderators may try to amplify social dynamics that increase comprehensibility and relevance, for example, through building a mutual mind map of work and health together with the participants (Jenny et al., 2020) and aligning the workshop actions with the team’s and organization’s structure, strategy, and culture (von Thiele Schwarz and Hasson, 2013). This may be complemented with a range of activating exercises, like “miracle questions” (i.e., asking participants to imagine they were asleep and as they wake up, all problems are gone – how would they notice?) and drawing together a vision of their future working conditions (Bauer and Jenny, 2018). Digitalization of interventions will further enhance the need for short scales to monitor the process and progress: Although workshops gain much of their impact through real-live interaction between human beings, some elements can be conducted virtually – or even must, when teams work remotely and/or at home. A short scale like the WASC may support both workshop moderators and teams to digitally check their journey, similar to the trend of “pulse surveys” to quickly check levels of stress and engagement in teams. Such digital (self-)monitoring may also help generate the amount of team-level process data that is needed to conduct reliable and valid evaluation studies of organizational health interventions. Hereby, short and generic scales could also support the linkage of collective process data with individual outcome data from a heterogenous range of teams and organizations.

## Data Availability Statement

The datasets generated for this study are available on request to the corresponding author.

## Ethics Statement

Ethical review and approval was not required for the study on human participants in accordance with the local legislation and institutional requirements. Written informed consent for participation was not required for this study in accordance with the national legislation and the institutional requirements.

## Author Contributions

AF, GB, and GJ conceived the study and collected the data. AF and GJ analyzed the data. AF, GB, and GJ led the writing. All authors contributed to the article and approved the submitted version.

## Conflict of Interest

The authors declare that the research was conducted in the absence of any commercial or financial relationships that could be construed as a potential conflict of interest.
